# Biographical accounts of the impact of fatigue in young people with sickle cell disease

**DOI:** 10.1111/1467-9566.13477

**Published:** 2022-04-29

**Authors:** Brenda Agyeiwaa Poku, Alison Pilnick

**Affiliations:** ^1^ School of Sociology and Social Policy University of Nottingham Nottingham UK

**Keywords:** biography, children, chronic illness, fatigue, qualitative methods, sickle cell disease, young people

## Abstract

Children and young people (CYP) with sickle cell disease (SCD) are a 'missing voice' in the debate on biography and sociology of chronic illness, meaning we know little about the social consequences of the illness for CYP. This paper examines the meaning of fatigue (a common symptom) for adolescents with SCD. Analysing 24 in‐depth interviews with adolescents aged 12–17 years in Ghana, we draw on the distinction proposed by Bury (1988) between 'meanings as significance' and 'meanings as consequence' to examine biographical aspects of fatigue. We argue that concepts of 'biographical disruption' and 'normal illness' do not easily accommodate the experience of CYP with congenital chronic illnesses like SCD, as their sense of (un)disruption and normality/continuity is contextualised relative to normative expectations about what it is to be a young person. At biographical transition points, illness/symptoms present from birth may evolve, shift and become experienced as 'new', 'different', or 'non‐normal'. They may become restrictive rather than continuous or disruptive. These experiences are influenced primarily by normative biographical expectations and the pursuit of identity affirmations. We propose that *biographical restriction*, *biographical enactment*, *biographical abandonment* and *biographical reframing* are more relevant concepts for understanding the experiences of CYP living with SCD.

## INTRODUCTION

Biographical disruption and related concepts have been widely applied as explanatory frameworks for understanding chronic illness in adulthood and childhood. Different normalisation concepts have subsequently been proposed to explain individuals' diverse responses to illness on their biography. But as of yet, no research has focussed on the biographical experiences of children and young people (CYP) with sickle cell disease (SCD) and/or has drawn attention to the ways culturally constructed understandings and conceptualisations of childhood/youthfulness and gender mediate their experiences and responses. This article examines biographical constructions among adolescents with SCD to address these gaps.

SCD is a chronic debilitating hereditary blood condition that disproportionately affects people of African descent (Piel et al., 2015). It is commonly characterised by chronic fatigue and unpredictable and episodic acute painful episodes/crises (Poku et al., 2018) that cause cumulative damage to multiple organ systems. Living with SCD can give rise to significant challenges, resulting from both symptoms and related psychosocial responses—stigma, shame, blame, social withdrawal and social exclusion (Atkin & Ahmad, 2001; Dyson et al., 2011, 2016; Poku et al., 2018, 2020). This article aims to develop new conceptual insights for understanding the biographical experiences of adolescents with SCD in Ghana regarding SCD‐related fatigue. Drawing on the distinction between *meanings as significance* and *meanings as consequence* proposed by Bury (1988), we argue that adolescents can experience what we term *biographical restriction* and employ *biographical enactment*, *abandonment* and *reframing* to overcome this restriction and develop and sustain their sense of biographical 'normality'. We will also show the importance of cultural contexts on these illness biographies.

### Biographical disruption and related concepts

The importance of biography in understanding chronic health conditions has long been recognised. The seminal concept in this field, Bury's (1982) *biographical disruption*, proposes a chronic illness as constituting a significant disruptive experience, which necessitates a fundamental re‐examination of one's self‐concept and biography. Following Bury, other researchers have coined expressions to highlight the significance of context for diversity in responses to chronic illness. These include *biographical disintegration* from parental shattered biographies after a son's suicide (Owen et al., 2008); *biographical abruption* relating to people diagnosed with motor neurone disease, who were found to lose all hope and experience the sudden breaking of their biography post‐diagnosis (Locock et al., 2009); and *biographical suspension*, in which individuals with nonspecific low‐back pain were seen to live a life ‘on hold’ (Bunzli et al., 2013, p. 913). However, the concept of biographical disruption has primarily been predicated on an adult‐centred model of illness, denoting the shift from a previously normal state of health to one of unexpected/unanticipated illness (Larsson & Grassman, 2012).

Consequently, there has been a debate about whether biographical disruption is a relevant concept for all those living with a chronic illness. Notably, Williams (2000:51) has argued that chronic illness may be accepted as 'biographically anticipated' 'normal illness' or 'normal crises' by the individual and thus not necessarily experienced as disruptive, using expressions such as *biographical continuity* and *biographical confirmation* to conceptualise this 'normal illness' phenomenon. He supported his argument with the earlier work of Carricaburu and Pierret (1995). They found that HIV‐positive men experienced *biographical reinforcement* of prior‐held components of identity built around homosexuality or haemophilia instead of experiencing disruption after a diagnosis. Several works have added weight to the 'normal illness' argument. For instance, Faircloth et al. (2004) coined the concept of *biographical flow* to explain how older people with comorbidities who experienced stroke viewed having a stroke as not a major disruption but as part of their ongoing biography. Findings from studies of illness experiences in older age have also suggested that a chronic illness might cease to be out of place or special in old age and thus may be experienced as more expected and less disruptive (Charles & Walters, 1998; Priestley, 2006; Sanders et al., 2002). On the other hand, other scholars have challenged this binary 'unanticipated and disruptive' or 'anticipated and normal' distinction. Larsson and Grassman (2012) argue that although many illnesses are anticipated in older age, this anticipation does not necessarily mean a lack of impact on the individual's life. Sanders et al. (2002) confirmed this by arguing how osteoarthritis can be simultaneously 'normal' as an inevitable part of old age and also highly disruptive.

Williams (2000) goes on to argue that a chronic condition experienced from birth or childhood may become a normalised part of everyday life for some people, suggesting the need for an extension of the biographical focus to ‘both ends of the life course’ (2000: 61). Some recent research has begun to explore the biographical impact of illness among CYP (Bray et al., 2014; Monaghan & Gabe, 2015; Polidano et al., 2020; Saunders, 2017), describing both the disruptive impact and normalised nature of chronic illnesses (Bray et al., 2014; Polidano et al., 2020; Saunders, 2017) whilst also describing the *biographical enrichment* and *renewal* aspects of medical interventions (Bray et al., 2014; Polidano et al., 2020). B*iographical contingency,* which describes how a chronic illness may be an 'only sometimes' problem for CYP, has also been proposed as an explanatory concept (Monaghan & Gabe, 2015). Interestingly, these child‐centred studies have highlighted how biographical experiences are influenced by a nexus of factors situated in the context of personal circumstances, such as social relationships and interconnectedness. These findings support Williams' (2000: 62) call for greater attention to the ‘timing, context and circumstances’ within which illnesses are 'normalised' or 'problematised' and how identities are threatened or affirmed as a result. Rather than focussing primarily on the destructive effects of illness, Williams calls for attention to be paid to the timing and context of chronic illness, notably the social variables, previous experience of hardship/traumatic events and the inter‐relationship between illness and an individual's key sources of identity.

Studying CYP's biographical experiences of illness as distinct from adults is particularly important due to the socially/culturally‐constructed expectations of the life stages they are required to transition into and embody. Saunders (2017), for example, highlights how his proposed concept of *recurrent biographical disruption* experienced by young adults with inflammatory bowel disease was more severe in young adulthood owing to the unique pressures and expectations of this life stage. This article will contribute to this emerging field and draw attention to cultural dimensions of illness experience, which are less commonly explored. We focus on how wider socially/culturally‐constructed understandings and conceptualisations of childhood/youthfulness and gender interact, and mediate the biographical experiences of adolescents with SCD in Ghana. We consider how they exercise their agency and competence in dealing with the impact of their symptoms on their biography within the boundaries of cultural conceptualisations/expectations. Within the broader Ghanaian culture, biographies of gender are an inextricable part of biographies of adolescence/youthfulness (Nkunya, 2003; Salm & Falola, 2002). While the latter is constituted around physical/social functioning and performance of culturally‐predetermined social roles/responsibilities (Nkunya, 2003; Salm & Falola, 2002), these roles are gender‐specific, with female roles/responsibilities centred around homemaking and that of males focussed on attaining leadership, professionalism and financial capacity (Adomako‐Ampofo, 2001; Nkunya, 2003; Salm & Falola, 2002).

## METHODS

The study reported here was conducted in 2015–2019 and aimed at exploring the fatigue experiences of adolescents with SCD in Ghana, using constructivist grounded theory (Charmaz, 2014). Work was informed by recognising that CYP are competent to speak for themselves about their experiences and perspectives on the social worlds in which they live (James et al., 1998). Although children's agency and competence are bound in intergenerational relations (Mayall, 1996), understanding children as social agents and co‐constructors of their social world is fundamental to studying their experiences and ways of dealing with health and wellbeing in everyday life (Brady et al., 2015). However, children's verbal skills, cognitive development and inherent power dynamics between adult researchers and children may influence the articulation of experiences (Poku et al., 2019).

### Sample and recruitment

The study was approved by two University ethics committees and the institutional review boards of two teaching hospitals. Twenty‐four (24) adolescents were recruited from two SCD treatment centres in Ghana. Adolescents were eligible for participation if they were aged 12–17 years, had no comorbidities and were not pregnant (to ensure that the data generated related to SCD‐related fatigue because fatigue is associated with many other conditions). The age range was based on the evidence on the onset of adolescence and pubertal development in children with SCD (Zemel et al., 2007) and the age when transferring responsibility for self‐management from parents to children begins (Nightingale et al., 2019). Clinicians at the clinics introduced the study to eligible adolescents and their parents. Those who expressed interest in the study were introduced to the first author, who provided them with detailed information about the research and what their involvement would entail. The first author was known to the clinicians as a service user.

**FIGURE 1:**
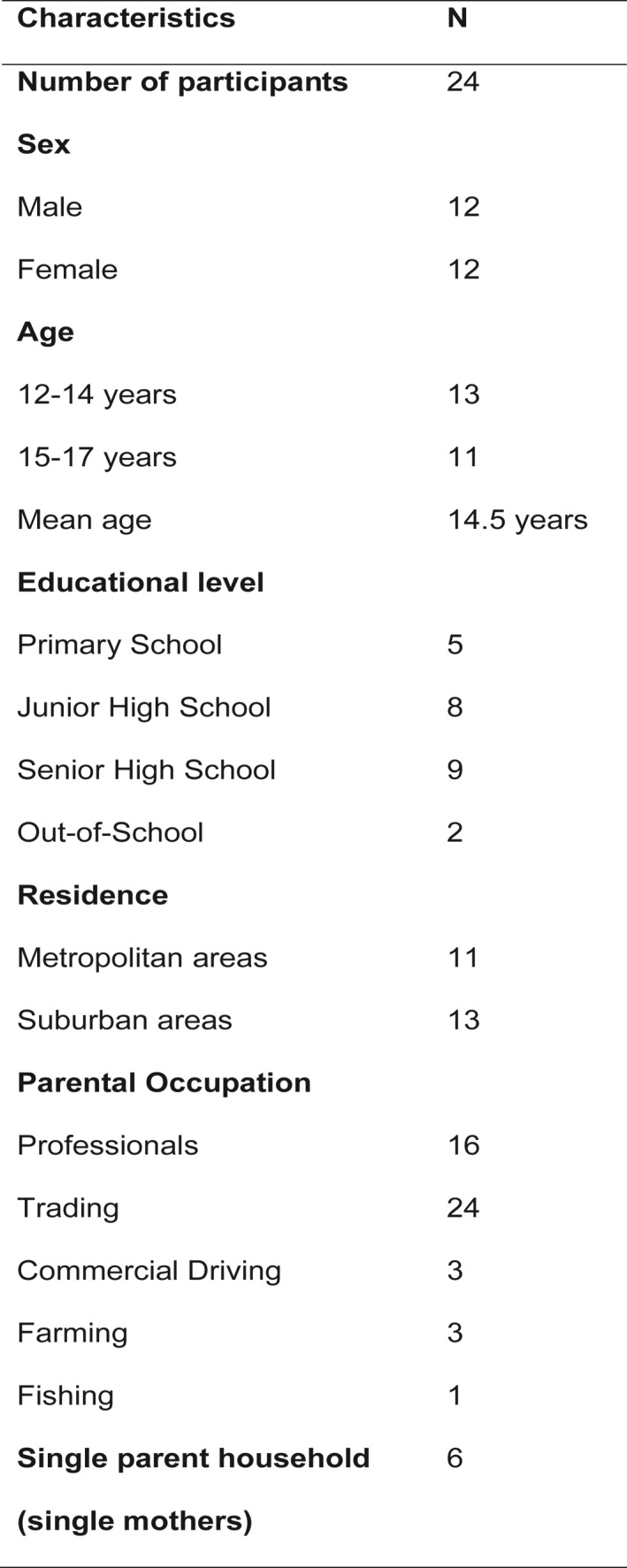
Participant demographic characteristics

Adolescents who continued to express interest gave their mobile phone numbers to the first author for her to contact them 2 days later. This researcher‐initiated contact was used to address any potential financial barriers to contact. After 48 h, the researcher phoned them to determine their decision and negotiated a scheduled interview session with those who agreed to participate in the study. At this point, verbal consent from the adolescents and verbal proxy consent from their parents were taken, and the consent forms were texted to them to review, whilst written consents were taken at the time of a scheduled interview. A purposive sampling approach was adopted to ensure maximum variation in sex, age, residence, educational level, and family socioeconomic background. Twenty‐four adolescents participated in the study (see Figure 1 for demographic characteristics).

### Data collection

A range of methods was adopted to allow the adolescents to convey their thoughts as capable social actors (Kellett & Ding, 2004), including conventional face‐to‐face semi‐structured interviews, drawings, photography and photo‐elicitation interviews. We aimed to engage the adolescents in forms of communications that were creative, flexible and responsive to their preferences and abilities (Poku et al., 2019) and that enabled them to construct rich narratives about their fatigue experiences. Participants chose the type of interview they wished to participate in, depending on convenience, preference, interests and abilities. Fifteen adolescents participated in the conventional interviews, and nine in the photo‐elicitation interviews. Interview venues and who joined them were based on the adolescent participants' preferences and convenience, with consent from their parents. Most of the interviews occurred in participants' homes (18/24), and the rest took place in their schools (2/24) or at the clinics (4/24). We endeavoured to conduct the interviews privately with the adolescent participants to support their free expressions of experiences and views; however, they were offered the choice of having an adult present during the interviews. Most of the interviews were conducted privately (22/24). Interviews were conducted in English and/or Twi and, where necessary, translated into English by the first author.

### Data analysis

Data were analysed in line with the principles outlined by Charmaz (2014), beginning with line‐by‐line coding of the first four interview transcripts. The codes generated were sorted into analytical/focussed codes. During coding, gaps identified in the data guided subsequent interviews (Charmaz, 2014). The focussed codes were used to analyse subsequent data. As coding continued, the focussed codes were refined, and new ones were generated as new threads for analysis became apparent. The focussed codes were subsequently compared and grouped into overarching categories and subcategories. The properties of the categories were identified, defined, related to each and refined further through data collection and coding to theorise the participants' fatigue experiences. The authors discussed the developing codes, categories, relationships, and interpretations and used reflexivity to determine how our experiences and insights about the phenomenon might have influenced the data analysis. Participants provided pseudonyms for anonymising their data and information.

## FINDINGS

Using the conceptual distinction proposed by Bury (1988), we examine the *meanings as significance* and the *meanings as consequences* of the fatigue experienced by the adolescents. The meanings were used to consider the complexities of the biographical accounts of the symptom. Previous sociological studies have highlighted the biographical importance of meanings assigned to illness and consequent action in dealing with symptoms (Bury, 1982; Sanders et al., 2002; Williams, 1984). In examining *meanings as significance*, we consider how participants present their fatigue as a normal part of their illness but a non‐normal and restrictive part of their biography as adolescents. We also consider the connotations of adolescence and fatigue in contemporary culture and how participants' biographical accounts make sense in the context of such connotations. We then follow this with an assessment of *meanings as consequence* to consider the biographical approaches fatigue evokes. Although we distinguish between these two types of meanings and present them separately, it is important to note that 'the experience of chronic illness always operates simultaneously at these two levels' (Bury, 1988, p. 92). We, therefore, consider how contrasting representations of SCD‐related fatigue as being both normal and non‐normal co‐exist and the implications of this simultaneous experience for the biographical approaches adolescents adopted to manage the symptom.

## MEANINGS AS SIGNIFICANCE

### Fatigue as a normal part of SCD

Most participants articulated their fatigue as a 'normal' and integral part of their illness. Merlin's (aged 14) and Ramisbeadz's (aged 15) accounts below are typical of the way they talked about the perceived causes of their fatigue:

I know I'm not able to do things like others because of my condition. One doctor explained to us that our red blood cells are not full like that of ordinary people. Ours have pinpointed ends, so they can't flow freely in the blood vessels, blocking them and causing pains. Ever since then, I got the understanding that because my red blood cells are not full, that's why I get tired quickly. (Merlin)

I think maybe because of the low haemoglobin caused by sickle cell, that is why I get tired quickly. (Ramisbeadz)

In the excerpts, both Merlin and Ramisbeadz appear to define their fatigue within the pathology of SCD, specifically linking fatigue to the distorted shape of sickle cells and the lower haemoglobin levels. They perceived and presented their fatigue as a biological (and thus objective) component of their illness. In addition to this, fatigue was viewed as being bound up with their biological bodies. In both accounts, the young people allude to a limited body, which accounts for a reduced performance capacity and physicality (see Poku et al., 2020 for a detailed discussion on fatigue, SCD and the biological body).

This connection between fatigue, SCD and the biological body led to an understanding shared by almost all the participants that fatigue is a norm (Poku et al., 2020). They offered a medical theory of causation, which readily objectified their fatigue. In the narratives, having limited energy and quickly running out of energy was typical with having SCD and thus a legitimate experience. However, while Bury (1982) proposes that the objectification of disease encourages health‐seeking behaviour, the contrary was observed in our participants. Most of the adolescents reported reluctance to seek treatment for their fatigue. When asked whether they had consulted their doctors about their fatigue, one commented, 'there's nothing anyone can do about it. Those who know the condition very well know that tiredness is part of it' (Christiano Ronaldo, age 16). For others, contact with and attitudes of doctors had reinforced the view that fatigue was an untreatable outcome of SCD and therefore a symptom they must endure, as exemplified by Shy Girl (aged 17):

One time, I complained to my doctor about it [tiredness], but all he said was that it's part of it [sickle cell] so rest when you feel tired and don't stress yourself too much. For me, tiredness brings the pain, but when I report to the hospital with pains, they just sort it [pain] out; they don't bother to ask about what triggered it. So, I don't talk or complain about it [tiredness] anymore.

In the accounts, fatigue is reported to be pervasive and significantly impacts young people's lives. Participants gave a medical explanation for their fatigue, although fatigue was not treated as a medical problem or something treatable by medical staff. These findings echo Sanders et al.'s (2002) study of participants with osteoarthritis who normalised their symptoms (painful joints and fatigue) and felt there was no effective way of treating them. While the views about the biological causes of SCD‐related fatigue offered the participants a sense of 'normality', it was not easy to reconcile with the 'non‐normality' of their everyday fatigue experience in adolescence.

### Fatigue as shifting, non‐normal and a biographical restriction in adolescence

Despite perceiving their fatigue as an inevitable and a normal consequence of SCD, at the same time, the participants articulated the fatigue they were experiencing as 'new', 'different' and 'non‐normal', as demonstrated by Atom (aged 16) and Miss Malaika (aged 15) in the extracts below:

When I was younger, I did nothing. My parents wouldn't let me do anything. I only played with toys or played video games with my siblings. The school bus took me to school and brought me home every day, and it dropped me off very close to the house, so I walked a very short distance, so I hardly got tired. When I do get tired doing something, I always thought it was normal to get tired . . . Even my father and big brothers complain of tiredness. I always saw my tiredness as normal. But, when I got to Junior High School, there was the pressure to do what everyone was doing. I started joining my friends to walk home after school even though my parents were still paying for me to join the school bus. Anytime I walk back with my friends, I felt very tired, which was not normal. (Atom)

Previously, I wasn't doing anything at all. My mother and my older sister did everything in the house. She only let me help with the cooking. Mum started introducing me to domestic chores when I was ten years old, in Primary 5, giving me my own chores. That was when I started feeling tired quickly and frequently. (Miss Malaika)

In the accounts, both participants narrate how the fatigue they are experiencing as adolescents is different from what they experienced in early childhood. They recount facing little difficulty with fatigue at younger ages due to parental control and restrictions or having little or no responsibilities and expectations. These offered them considerable protection. Indeed, Atom describes the fatigue he experienced in early childhood as 'normal' and comparable to that of his father and older siblings. Similar findings have been reported among younger children with SCD and thalassaemia in the UK, where family and parental support have positively impacted a child with SCD's illness experience (Atkin & Ahmad, 2001).

However, according to Atom and Miss Malaika, as they grew older, they were confronted with emerging expectations, norms, and responsibilities of adolescence, which accounted for a new, different, non‐normal fatigue. For Atom, increasing peer relationships and social interactions changed his experience of fatigue. Miss Malaika's new fatigue was associated with her new gendered domestic responsibilities. (for further discussion of the gendered dynamics of parental care, support and expectations, see Poku et al., 2022). These quotes illustrate how the expectations and pressures of adolescence caused a change in the nature of the fatigue the adolescents experienced, making it a different experience from that of their peers. Christiano Ronaldo's narrative succinctly captures this:

There's a difference between my tiredness and that of my friends and siblings. For instance, when we are playing football in school, and someone complains of tiredness, they keep playing, but for me, if I'm tired, I can't continue playing. I must leave the game and find somewhere to sit and rest. My tiredness is twice theirs.

The occurrence of fatigue is not 'new', but the way the adolescents experience it is different. This is related to their changing roles and expectations—both ones they place on themselves and ones which others place on them. Consequently, with a transition to adolescence, the experience of fatigue shifts and evolves from 'normal' and undisruptive to 'non‐normal' and restrictive. This experience is closely linked to the taken‐for‐granted activities and expectations of adolescence, as is also found in the illness experiences reported among older children with SCD and thalassaemia in the UK (Atkin & Ahmad, 2001). However, for our Ghanaian participants, parental and societal expectations around the contributions that adolescents make in the home and within the community are more entrenched, and therefore, this has more of an impact. Consequently, the culturally‐constructed standards and expectations of adolescence significantly disrupted their fatigue (illness) experience.

### Biographical trajectories as illness disrupters

While, according to the adolescents, the cause of fatigue is objective, how it is experienced is subjective. This provides a social model of causation of fatigue in adolescence. The participants highlighted how they felt social expectations, responsibilities and relationships of adolescence contributed to their fatigue experience. It can be said that while the medical model of causation signified the 'normality' and legitimacy of fatigue, the social model of causation provided them with a framework to make sense of their fatigue. However, the process of transition to adolescence ruptured the adolescents' existing system of meanings, requiring them to redefine their understandings (Zittoun, 2006) and resulting in a shift from a sense of 'normality' to 'non‐normality' during transition.

As Bury (1982) noted, illness narratives often begin with a description of a period prior to onset and diagnosis before explaining the disruptive impact of the illness and consequential deviation from the individual's normal functioning and life. However, for children born with chronic illnesses, there is often an assumption that the presence of the illness encourages its normalisation and biographical continuity (Williams, 2000; Williams et al., 2009). By contrast, although our participants offered a personal and self‐referential narrative of their fatigue experience, they indicated change and the onset of a recognition of a personal non‐normality, suggesting the beginning of disruption. The disruption the participants described was a kind not necessitated by their illness or its symptom (fatigue), as Bury (1982) suggests, but rather a disruption caused by a normative biographical trajectory.

Thus, the transition between different life stages can disrupt the normalisation that has been (actively) achieved up until that point. As the participants have always experienced fatigue, it is not a biographical disruption in Bury's sense; however, its impact becomes more significant at this transition point. Therefore, it begins to be experienced as a biographical restriction. Similarly, fatigue does not become normalised in adolescence or experienced as a biographical continuity in Williams' (2000) sense. Instead, it shifts so that it is experienced as non‐normal, discontinuous, and disrupted by normative biographical trajectories. This 'non‐normality' is constructed by participants with reference to peers and siblings and broader medico‐social perceptions of symptoms and 'age‐appropriate' normal development.

### Cultural connotations of adolescence and fatigue

The meaning and significance participants attributed to their fatigue were grounded in wider societal perceptions of adolescence. As Backett‐Milburn (2000) argues, bodies of CYP are generally perceived and expected to grow to withstand physical extremes and embody stamina and strength. A 'healthy' or 'normal body' in childhood/adolescence tends to be equated with physical and social activeness. These medico‐social conceptions and age norms are used to predict, understand and control development in CYP (Habermas, 2007; McLaughlin & Coleman‐Fountain, 2014). Consequently, children and adolescents' inability to engage in physical and social activities and their settling for less physically‐demanding activities or resting during the day can be construed as unnatural and not 'normal' (Backett‐Milburn, 2000; Monaghan & Gabe, 2015). In the specific case of fatigue, it is generally perceived as antithetical to youthfulness/adolescence (Monaghan & Gabe, 2015).

Given these pervasive cultural connotations of adolescence and fatigue, it is unsurprising that they were present in the participants' stories and employed in narrating the meaning of fatigue. This aligns with Zittoun's (2006) argument that cultural products act as symbolic resources in young people's development. Barbie's (aged 13) and Merlin's (aged 14) accounts below demonstrate this:

In the house and among my friends, it's like you're not supposed to complain that you're tired when everybody isn't. It's like if you do something small and you say you're tired, it means you don't want to do it, or you're lazy. They make it sound like you have decided to be tired. They don't believe you because how can you be tired when you've almost done nothing and when you're not disabled or an older person who is weak and frail. (Barbie)

It [tiredness] makes me feel like an old man like. . . My body is some aged machine not as fit as that of my peers because I can't stand those running, jumping and ball games. I do little, and I'm exhausted. It makes me feel bad because they tease me that I'm weak, girly, and there's no fun in playing with me because I'm always complaining that I'm tired. (Merlin)

Barbie's excerpt above highlights that reduced functionality and physicality are perceived as synonymous with old age and physical disability in her social domains. This was also a perception held by Merlin, who talks about how fatigue makes him feel like an older man and describes his body as an aged machine. This perception of fatigue as a norm for older people is illustrated by Sanders et al. (2002), whose older participants with osteoarthritis perceived their illness and its symptoms as a commonplace and normal aspect of the biography of older age. The fact that young people with SCD do not observably fit into groups where fatigue is accepted leads to additional consequences. Barbie explains how the invisibility of her illness, her 'normative' appearance and her young age cause others to discredit her fatigue, with the result that, although it is a legitimate consequence of her condition, it is perceived as illegitimate by others. Both Merlin and Barbie talk about the risk of being stigmatised as 'weak' and 'lazy' when they fail to present their bodies as 'youthful', and their fatigue becomes public knowledge. This results in double stigmatisation since, according to Newton et al. (2013), being disbelieved or discredited is stigmatising in itself. Fatigue, therefore, restricted not only their activities but also their biographical presentations, leading to significant social consequences.

## MEANINGS AS CONSEQUENCE

### Biographical approaches to the restriction of fatigue

The adolescents participating in our study reported employing multiple and changing strategies to deal with fatigue's actual and potential biographical restriction and its stigmatisation, showing how the meanings they attached to their illness had consequences for their lives. These will be discussed using the concepts of *biographical enactment*, *biographical abandonment* and *biographical reframing*.

### Biographical enactment

All the adolescents attempted to portray themselves according to the expected biography of adolescence and youthful bodies. There were internal and external drives to attain a 'normal' presentation and present a 'healthy' young body image, as Joe Mettle describes below:

Being able to push myself to do something makes me happy even when I end up with severe painful tiredness. It makes me feel like everyone else and relevant.

Here, Joe Mettle talks about the importance of overcoming fatigue's biographical restrictions by pushing beyond his body's limits, despite the difficult consequences he knows will result. His talk shows a moral dimension to biographical enactment: adolescents who can successfully demonstrate accepted biography often describe feeling happy, content and satisfied (Balfe, 2009; Lupton, 1996) given their prominence in Ghanaian culture. Enacting adolescence and youthfulness through physical and social activity was a source of self‐satisfaction for most adolescents in this study. However, this was particularly marked for the male participants due to the importance of high physicality and functionality in traditional constructions of masculinity (Connell, 1995; Wedgwood et al., 2020). Indeed, Atom explicitly defines masculinity to include traditional assumptions of male identity such as high physical performance, physical resilience, power and bravery:

When I think of a man, what comes to mind is fitness, strength and energy levels.

Therefore, the biographical restriction of fatigue shrinks the male participants' masculine biography. In Joe Mettle and Atom's accounts, biographical enactment was an important strategy for their masculine affirmation and self‐esteem. It also enabled the adolescents to fit in with peers and secure their place in their peer groups (Balfe, 2009), as depicted by Hazzard:

When I'm with my friends and playing football, I don't want to disappoint the team; the rest count on me to play my position. When you commit yourself to something, you've to make sure you complete it. If I disappoint them, they'll never let me play again, so I must show they can constantly rely on me. That makes me force myself even when I feel tired until I become very tired and can't continue.

In this excerpt, Hazzard explains the importance of biographical enactment to his social‐ and self‐presentation, social acceptance and inclusion, and sense of belonging. He talks about it helping him to develop and present an acceptable group identity. It allows him to receive 'empirical validation' (i.e., being perceived as reliable and trustworthy) from others, legitimising that he is 'normal' and accepted (Charmaz, 1983, p. 170). However, this biographical approach to countering the biographical restriction of SCD‐related fatigue comes at a high cost to the adolescents. While it enables them to conform to the expected biography of adolescence, it has a limit, which Hazzard acknowledges. Indeed, in both Joe Mettle's and Hazzard's accounts, severe fatigue and SCD‐related pains signify the limits of biographical enactment. Thus, biographical enactment means subordinating one's health so as not to let oneself and others down. For Joe Mettle and other participants, this was a cost they were willing to pay for a 'normal' presentation and peer acceptance.

Aside from the social domain of friendship and peer groups, biographical enactment to meet expectations was also employed by the female adolescents in the home context, despite the health implications. For the female participants, gender enactment was central to biographical enactment, as Mascara Lady (aged 13) illustrates:

I get tired when I'm washing the utensils in the house, so I stop and take some rest and continue afterwards. But when I stop and rest, my mum gets angry and shouts at me to hurry up and finish the dishes. When she shouts, I get angry because she is putting pressure on me. But when my mum is angry with me, it makes me sad, so I try and do things the way she wants even if I'm tired.

Mascara Lady's account, together with Hazzard's above, underscores the fact that biographical enactment was always employed in relation to others, and particularly in relation to central places, events, activities and experiences of social life where the participants felt 'on display' (Balfe, 2009; Poku et al., 2020). Buchbinder (1994) argued that biographies are publicly enacted and require others' approval. It is, therefore, unsurprising that biographical enactment frequently appeared in the narratives and that participants describe its daily use. Despite the daily attempts to affirm their identity by constantly pushing themselves to enact biographical expectations, biographical enactment ultimately threatened the participants' health and risked or undermined their identity affirmation. This forced them into employing another biographical approach we have termed biographical abandonment.

### Biographical abandonment

Unlike Joe Mettle above, who describes how continuing to push himself physically makes him happy, some adolescents could not continue with biographical enactment due to the health implications it evoked for them. This led to biographical abandonment, that is, the young people abandoning the active pursuit of conventional adolescence and gender biographies. This scenario is described by Lawrence (aged 16) below:

When I'm tired, I feel I need to continue because I want to show I'm also normal, which leads to whole‐body tiredness, and when I begin to rest, I feel pains in my back and limbs. I have had whole‐body tiredness that led to a severe painful crisis. In 2015, I came back home from school; I was very tired … you know the whole‐body tiredness, so I came home in a taxi . . . I went to rest, and I fell asleep. When I woke up, I couldn't walk. I was having very terrible pains, so I was admitted to the hospital. I was admitted for two months two weeks. I missed the whole school term. That whole‐body tiredness is what I'm scared of every day, but it's so hard to avoid it with everything I'm expected to do. I have realised that I can't be like my peers and friends no matter how much I push myself. So, I have decided to cut down on spending time with my friends and engaging less in physically‐demanding activities with them. I keep to myself most times. But it is hard and sad to accept this and not want to be like others or not want others to see you as normal.

Here, Lawrence recounts a life‐threatening consequence he experienced following his attempts at biographical enactment. His attempt to enact the conventional biography of adolescence and youthfulness triggered a 'whole‐body tiredness', which subsequently resulted in severe SCD‐related pains, for which he was admitted to hospital for an extended period. Importantly, this biographical enactment was not carried out in ignorance of the possible consequences, as Lawrence's account highlights his knowledge of the importance of rest. However, at the same time, his awareness of the normative expectations of what it is to be a young person led him to persist with biographical enactment until the life‐threatening episode. Lawrence's account also shows how paradoxically, because of the nature of SCD, attempts at biographical enactment run the risk of highlighting rather than masking the difference between the young person and their peers—by trying so hard to be 'normal', they eventually and inevitably undermine that 'normality'.

According to Lawrence, his episode of severe illness forced him to recognise the unsustainability of biographical enactment. Consequently, he talks about an active and intentional abandonment of the pursuit of the conventional biography of adolescence. For him, biographical abandonment involves reduced peer engagements, reduced engagement in physical activities, and self‐isolation; while it is health‐protective, it comes with other significant costs. Lawrence's last statement indicates how hard a decision of biographical abandonment is to a young person, given the significant implications it carries for both self and others' perceptions.

Biographical abandonment was a common strategy among the participating adolescents despite these potential social costs. However, they reported selectively employing it in social domains or contexts where they had greater autonomy and agency, like peer relationships and interactions. Biographical abandonment was not used in academic engagements or relational situations with adults. In these contexts, biographical enactment was necessary due to the participants' perceptions of people's lack of understanding regarding SCD and the marked power differentials in adult‐child relational contexts. Importantly, selective biographical abandonment in the context of peer interaction meant more energy and strength reserved for biographical enactment in these other contexts. Consequently, biographical enactment and abandonment co‐existed for the majority of the participants.

Selective biographical abandonment also bought the adolescents time and energy to invest in their academic work and education. Education and intellectual careers were seen as means to control the future risk that the biographical restriction of SCD‐related fatigue would pose in adulthood. For the female participants, like Ramisbeadz, academic excellence and an intellectual career in the future were presented to afford them the luxury of domestic support to meet the traditional Ghanaian female biography in adulthood. Noteworthy is that all the female participants acknowledged the importance of education and future careers. However, this acknowledgement did not seem to indicate them explicitly challenging gender (domestic) expectations. Instead, there was evidence of explicit acceptance of gendered expectations by some participants, and future careers were viewed as opportunities to have economic, social, and material capital to support the enactment of gender roles. They, therefore, imagined the future in terms of which aspects of the likely biographies they foresaw being able to enact or abandon, leading to a strategic focus on abandonment now in order to enable enactment later:

I look forward to being a successful paediatrician, a wife and a mother in the future. When I think about my fatigue and the goals I've set for myself, I feel overwhelmed, but I believe if I achieve the career first, the other roles will be easy to manage … you know … I'll be respected; I can afford things that will make chores easier for me, you know, like a housemaid, washing machine, rice cooker, dishwasher, well‐furnished house, it'll then be easy to be a wife and mother and be normal (Ramisbeadz).

In the account, Ramisbeadz describes her future aspiration of fulfiling the biographical expectations of an adult female—a wife and mother. She highlights her strategy for achieving them, which involves first having a successful medical career. According to her, this will provide her with the resources/capital to help make the aspects of traditional roles that she will not abandon easier to enact in the future.

Like the females, the male participants also felt education and becoming members of intellectual professions would allow them to reproduce the conventional male biography in adulthood. They anticipated that intellectual careers would protect them from the harsh realities of physically‐demanding jobs and enable them to navigate biographical expectations of being an adult male—a husband and father—despite their fatigue. Aligning with other male participants and including them in his account, Joe Mettle expressed the significance of education and intellectual careers to the enactment of manhood for males with SCD:

I think a man is someone who fulfils his responsibilities by providing for those under his care … his children, wife, and parents. But as for us sickle cell people, we don't have the energy and strength to do hard work. We've to go by our energy limits, so we need to choose jobs within our energy limits to take care of our families in the future. That's why I want to be an architect. I have chosen architecture because of my energy level. A man also needs to be physically strong to do any work to look after his family, but as for sickle cell people, we don't have the strength and therefore can't do any work. We need to consider our energy limits. If you're a sickle cell guy and manage to take care of yourself and your family, you're a man even though we're not strong.

Like Joe Mettle and Ramisbeadz, almost all the participants expressed career choices based on their physical limitations and not their strengths or interests, showing anticipation that focussing on intellectual work was their only guarantee for 'normal' adulthood. They perceived that the selective biographical abandonment of other aspects of adolescence to enact academic aspects offered them the best opportunity to work towards 'normal' adulthood. So far, we have presented the biographical approaches—enactment and abandonment—that occurred commonly in our data. Finally, we draw attention to a different approach that occurred in one case.

### Biographical reframing

As shown through the data extracts reproduced above, most participants in this study worked to enact or abandon aspects of biographies conventional to the Ghanaian society. However, one adolescent engaged in what we have called biographical reframing. Henry Ford's (aged 12) account showed an acute awareness of these conventional biographies, but rather than rejecting his own ability to fulfil aspects of them, instead, he rejected the biographical trajectory itself and provided a re‐conceptualisation of this:

I don't see myself as weak because sometimes I do things my friends can't do. I can draw, and some of them don't know how to draw. I can sing, but most of the boys can't sing. It's only most of the girls that can sing. So, I'm strong. Those who say I'm weak don't know me very well, so I don't think they are correct because I can do so many things that they can't. I don't think being strong is all about running or playing for long but being able to do things like drawing, riding a bicycle, and singing. They all show that I'm strong. If I can't run and play like my friends because I get tired quickly, I'm still normal and not different because there're other things that I can do that others can't do. Nobody can do everything. If you're only normal when you can do everything, then nobody is normal in the world because nobody can do everything.

Here, Henry Ford shows an awareness of hegemonic masculinity and its emphasis on physical and bodily performance (Connell, 1995) but redefines strength and 'normality' unconventionally. He accepts the concept of strength but instead recognises that it is not always about physical performance and resilience but can be shown in artistic and creative ways. He also highlights the fact that people differ in strengths and capabilities. Rather than attempting to replicate the strengths of others despite his physical limitations, he focuses on his artistic and creative abilities. This biographical reframing caused him to view himself positively despite the biographical restrictions SCD‐related fatigue imposed on him.

In addition, Henry Ford's positive self‐regard and recognition of his strengths gave him the confidence to pursue an unconventional biography informed by his own capabilities rather than the conventional biographies of adolescence and masculinity. As he describes:

I draw instead of playing games and running. That way, I don't use so much energy, so I won't get tired. That's why I love drawing. I have been drawing since I was small. I'm famous in school because of my drawing. When my friends are playing, I sit and draw them and show them afterwards. It's not like being athletic … playing and running, and being popular because of it as a boy … but it saves me from getting very tired and sick.

In this account, Henry's pursuit of unconventional biographies did not mean reduced peer interactions or self‐isolation. Instead, he talks about integrating his unconventional activities into the conventional activities of his peers. He also hints at how focussing on his artistic strength and excelling in it offered him a social status comparable to high physicality among his peers but without detrimental consequences for his health. Thus, for this participant, reframing conventional biographies to incorporate his strengths boosted his self‐esteem and confidence in effectively negotiating social relationships, interactions and norms without subordinating his health. Indeed, the biographical restriction of fatigue had a positive outcome for this young person through the construction of non‐hegemonic masculinity (Wedgwood, 2013) and enabling him to subvert social structures to demonstrate agency (Bury, 2002).

## CONCLUSION

We have used Bury's (1988) conceptual distinction between *meanings as significance* and *meanings as consequence* to articulate the representations of the meaning of fatigue amongst adolescents with SCD in Ghana and consider the complexities of the young people's biographical accounts of the symptom. We have shown two distinct representations of how adolescents with SCD experienced fatigue and the meaning of that experience. First, as a legitimate aspect of their illness, it was seen as normal, integral and inevitable that they should experience fatigue. Fatigue was also experienced as 'normal' and undisruptive in early childhood. On the other hand, fatigue shifted to 'non‐normal' and restrictive in adolescence, despite its presence from birth. These accounts of 'normal and undisruptive' and 'non‐normal and restrictive' co‐exist in the biographical narratives of the young people and make sense in the context of cultural connotations of adolescence and youthfulness.

We found that having SCD was not reported as the primary influence on the experience of fatigue and that time spent with the illness did not make it easier or harder for them to confront the symptom (cf Larsson & Grassman, 2012). Biographical expectations and the cultural connotations and significance of adolescence shaped the young people's illness experience. Indeed, biographical trajectories were perceived and experienced as disruptive of illness experience. Notably, while fatigue was experienced as 'normal' and undisruptive in childhood due to greater parental control and support, it was not experienced this way in adolescence. Instead, it was experienced as a biographical restriction to the normative biography of adolescence and youthfulness, with a future potential to restrict 'normal' adulthood.

Thus, notions of biographical disruption and normal illness do not easily accommodate the experience of CYP with SCD, as their sense of (un)disruption and normality/continuity is contextualised relative to normative expectations about what it is to be a young person. While biographical disruption is principally focussed on how the onset of chronic illness disrupts what has been established (Bury, 1982), the concept of normal illness suggests that the presence of a condition from birth results in its normalisation (Williams, 2000). However, our study found that for CYP with SCD, these are reversed: (1) it is transition processes (e.g., adolescence) and not the illness that disrupt the sense of 'normality' and biography that has previously been established with regard to the illness and its symptoms during earlier childhood, and (2) biographical trajectories and transition processes cause illness/symptom experiences to evolve, shift and become disrupted.

Our data also shows that the biographical restriction of fatigue threatened the young peoples' biographical identities. Consequently, they employed various approaches to coping with this. Most attempted to affirm their identity via what we have called biographical enactment, constantly pushing themselves to enact biographical expectations to sustain, actualise and secure 'normal' presentations and identities (Giddens, 1991) to avoid biographical disruption (Bury, 1982) and what Frost (2005) calls identity damage. However, due to the nature of SCD, biographical enactment was threatening to the participants, risking or undermining identity affirmation due to the inevitable health consequences. As such, respondents described being forced into biographical abandonment; this approach co‐existed with biographical enactment. Participants chose which one to deploy depending on the time, context and circumstance and the need to balance identity affirmation and health maintenance. The presentation of biographical abandonment was also oriented both toward the present and the future in terms of how participants anticipated aspects that could be abandoned would shift over time. In only one instance in our data, a participant engaged in biographical reframing to help them pursue socially acceptable alternatives to conventional biographical 'normality'.

This is the first study to provide an in‐depth examination of the biographical perspectives of young people with SCD. It develops the concept of biography by stressing that young people born with a chronic illness like SCD (1) experience different biographical changes to adults due to the temporality and fragility of identity and independence which characterise life transitions, particularly in adolescence and emerging adulthood; (2) may experience symptoms present at birth differently at transitions due to changing biographical expectations; and (3) use diverse and changing biographical approaches—*biographical enactment, abandonment and reframing*—to balance growing up, the affirmation of biographical identities, and illness control. In addition, the analysis presented here underlines the importance of cultural contexts in understanding illness biographies. We have shown how culture mediates which aspects of biography are seen as open to negotiation in terms of enactment, abandonment and reframing. In this way, this article responds to the call to consider the appropriateness and reach of the biographical concepts of continuity and disruption in a more refined contextualisation (Larsson & Grassman, 2012).

## CONFLICT OF INTEREST

The authors declared no potential conflicts of interest concerning the research, authorship, and/or publication of this article.

## AUTHOR CONTRIBUTIONS

**Brenda Agyeiwaa Poku:** Conceptualisation (Lead); Formal analysis (Equal); Funding acquisition (Lead); Writing – original draft (Lead); Writing – review & editing (Equal). **Alison Pilnick:** Conceptualisation (Supporting); Formal analysis (Equal); Funding acquisition (Supporting); Writing‐ original draft (Supporting); Writing – review & editing (Equal).

## Data Availability

The data that support the findings of this study are available on request from the corresponding author. The data are not publicly available due to privacy or ethical restrictions.
